# Antigen-Specific Monoclonal Antibodies Isolated from B Cells Expressing Constitutively Active STAT5

**DOI:** 10.1371/journal.pone.0017189

**Published:** 2011-04-15

**Authors:** Ferenc A. Scheeren, Caroline M. M. van Geelen, Etsuko Yasuda, Hergen Spits, Tim Beaumont

**Affiliations:** 1 Department of Cell Biology and Histology, Academic Medical Center, University of Amsterdam, Amsterdam, The Netherlands; 2 AIMM Therapeutics, Academic Medical Center, University of Amsterdam, Amsterdam, The Netherlands; 3 Tytgat Institute, Academic Medical Center, University of Amsterdam, Amsterdam, The Netherlands; New York University, United States of America

## Abstract

**Background:**

Fully human monoclonal antibodies directed against specific pathogens have a high therapeutic potential, but are difficult to generate.

**Methodology/Principal Findings:**

Memory B cells were immortalized by expressing an inducible active mutant of the transcription factor Signal Transducer and Activator of Transcription 5 (STAT5). Active STAT5 inhibits the differentiation of B cells while increasing their replicative life span. We obtained cloned B cell lines, which produced antibodies in the presence of interleukin 21 after turning off STAT5. We used this method to obtain monoclonal antibodies against the model antigen tetanus toxin.

**Conclusions/Significance:**

Here we describe a novel and relatively simple method of immortalizing antigen-specific human B cells for isolation of human monoclonal antibodies. These results show that STAT5 overexpression can be employed to isolate antigen specific antibodies from human memory B cells.

## Introduction

In general, vaccination is a safe and efficient way to protect the human body against specific pathogens. However, vaccination is only applicable as a preventive measure, and development of new vaccines is a slow and expensive process. As an alternative, the use of sera enriched for pathogen-specific antibodies has been suggested [1], [2]. Treatment with pathogen-specific antibodies could then be applied in a prophylactic as well as in a therapeutic setting. However, non-human derived sera often provoke an immune response, thereby limiting the maximum number of treatments. Other possible caveats are the fact that it is difficult to obtain large amounts of sera of the same quality, and the risk of contamination with pathogens, in particular with viruses such as but not limited to Human Immunodeficiency Virus (HIV) and Hepatitis C Virus (HCV).

An alternative is the use of monoclonal antibodies [3]. Mouse monoclonal antibodies such as OKT3 have been used to treat humans but with limited success due to the immune response these antibodies provoked. An alterative approach could be the use of fully human antibodies. For this, novel technologies have been developed, including humanization of mouse antibodies, phage display of human B cell libraries, single cell PCR technologies and the creation of mice that express human immunoglobulin genes [4], [5], [6], [7], [8], [9].

All of these technologies have resulted in clinical relevant antibodies but most methods do not directly tap the potential of the human immune system. Indeed, the human immune system itself can safely be assumed to be the best in generating highly efficacious antibodies and these antibodies are most likely superior to those generated from mice or using phage display. In addition, such antibodies may have a better safety profile than antibodies derived from mice. Novel technologies to obtain monoclonal antibodies from human B cells include EBV transformation of antibody-producing B cells activated by TLR9-agonists [10], [11] and single cell PCR to obtain immunoglobulin genes from individual *ex vivo* isolated B cells [12], [13]. We have previously shown that with forced expression of BCL-6 in human B cells stable human monoclonal antibody secreting cell lines can be produced [14], [15]. We moved on to describe that ectopic expression of a constitutively active mutant of the transcription factor Signal Transducer of Transcription 5 (STAT5) in human memory B cells resulted in a differentiation block of activated B cells, preventing them to mature into plasma cells. STAT5 transduced cells resemble activated germinal center centrocytes and show enhanced survival and expansion [16].

In the present paper we exploited this enhanced survival and expansion of human memory B cells that express an active form of STAT5 in order to obtain antigen specific immunoglobulin. We established a series of cloned lines of human B cells that expressed an inducible STAT5 construct. By turning off STAT5 the clones regained their capacity to produce antibodies allowing identification of clones that produced specific antibodies.

## Results

### STAT5bER^pos^ B cells preserve capacity to produce immunoglobulins

**Figure 1:**
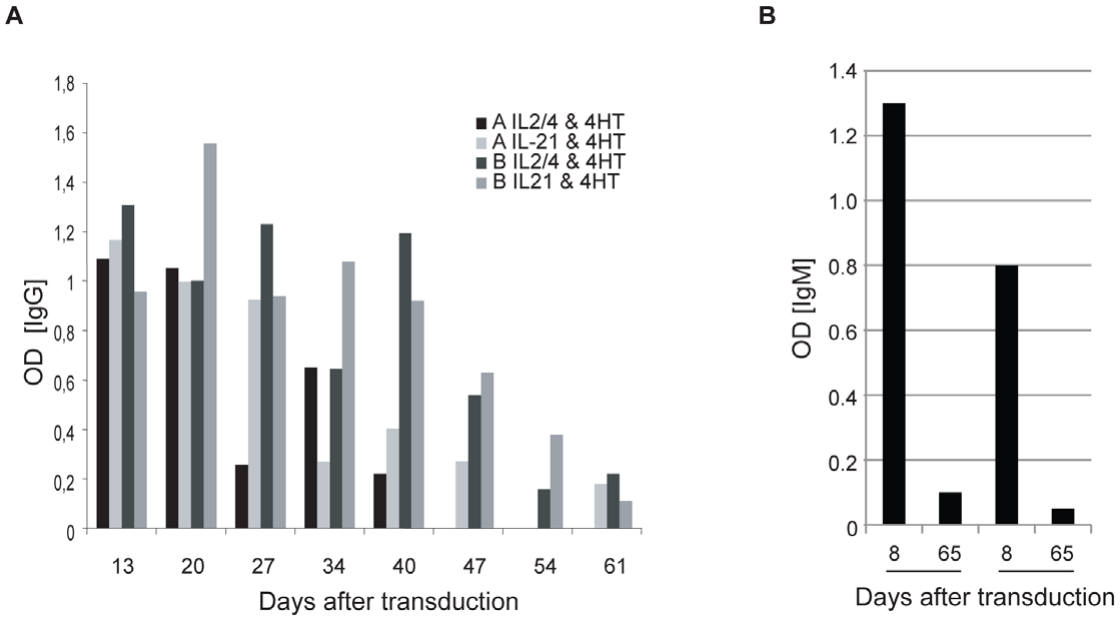
STAT5bER immortalized memory B cells down regulate Ig secretion. A) IgG production in time from STAT5bER transduced memory B cells cultured in the presences of CD40L, IL-2, IL-4 and tamoxifen (4HT) or CD40L, IL-21 and tamoxifen (4HT). Two representative donors out of 10 donors are shown. B) IgM production of peripheral blood B cells transduced with CASTAT5b cultured in the presence of CD40L, IL-2 and IL-4 at day 8 and 65 after transduction. Depicted are data from two donors.

Previously we have published that ectopic expression of active STAT5 mutants in human primary B cells results in a block in B cell differentiation and that these cells show enhanced survival and expansion [16]. We extended these findings by showing that constitutive activation of STAT5 in B cells led to loss of antibody surface expression when cultures were maintained for more than 6 weeks in the presence of IL-2 and IL-4 [17]. We then investigated whether we could exploit the immortalizing capacity of active STAT5 mutants in order to obtain human monoclonal B cell lines, which secrete antigen specific antibodies. For this, peripheral blood CD27^pos^ memory B cells were cultured in the presence of irradiated CD40-Ligand expressing L cells (CD40L-L cells) and interleukin (IL)-21 prior to retroviral transduction with constitutively activated (CA) STAT5b (CA-STAT5b). Pre-stimulation with IL-21 induced strong proliferation, which resulted in transduction efficiencies of 40 to 80% [15], [18]. Following transduction, in-active CA-STAT5b is expressed as a chimera with the hormone-binding portion of the Estrogen Receptor (ER) (CA-STAT5bER) [16], thereby STAT5b is expressed in the cytoplasm complexed by heat-shock proteins. Incubation with tamoxifen (4-HT) leads to dissociation of the heat shock proteins from constitutively active STAT5bER, which results in receptor dimerization and subsequent translocation to the nucleus where it initiates STAT5 specific transcription [16], [19]. First we studied the effect of STAT5 transduced cells cultured with IL-2, IL-4 and tamoxifen or with IL-21 and tamoxifen. IL-2 and IL-4 induce proliferation of human primary B cells similar to IL-21 but IL-21 has been shown to also induce strong differentiation of B cells [20], [21], [22], [23], [24], [25]. The secreted immunoglobulin levels in the supernatant of the CA-STAT5bER B cells were measured over time. Around 3 to 4 weeks after transduction the level of secreted IgG started to decrease for the tamoxifen/IL-2 plus IL-4 and tamoxifen/IL-21 cultured cells (Figure 1a). We also determined the long-term effect of constitutively active STAT5 expression on IgM secretion. IgM expressing peripheral blood B cells were transduced with CA-STAT5bER and cultured with tamoxifen, IL-2 and IL-4. Similar to the IgG secretion levels, IgM secretion disappeared in CA-STAT5bER B cells after prolonged culture (Figure 1b).

**Figure 2:**
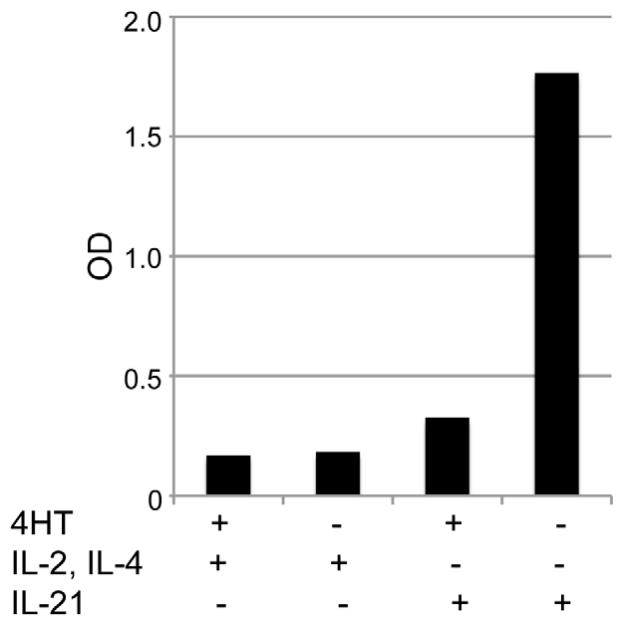
IL-21 induces enhanced antibody secretion by CA-STAT5bER immortalized memory B cells. Polyclonal CA-STAT5bER transduced memory B cells were deprived from tamoxifen (4HT) for 7 days and cultured with IL-2 and IL-4 or with IL-21 before IgG secretion was determined. Data shown are from one representative donor out of 5 donors. In all cultures (in culture for at least 2 months) transduced B cells had lost surface immunoglobulin expression.

The decline in immunoglobulin secretion corresponded with a decline in surface immunoglobulin expression [17]. This shows that with time, constitutive expression of STAT5 in human B cells leads to diminished expression and secretion of immunoglobulins. We questioned whether these effects could be reversed by de-activation of constitutively active STAT5 through removal of tamoxifen from the culture. We cultured the cells with tamoxifen, IL-2 and IL-4 for weeks to months until immunoglobulin production had vanished and then removed tamoxifen from the culture. In these cultures, when treated with IL-2 and IL-4 immunoglobulin secretion remained low or absent (Figure 2), but when they were treated with IL-21, IgM or IgG immunoglobulin secretion resumed (Figure 2 and Figure S1). The immunoglobulin secretion lasted for about 7 days (Figure S2). After approximately 14 days antibody production came to an end and the transduced B cells died [16]. It has been shown previously that IL-21 induces differentiation of B cells into plasma cells, but this was not observed in our B cells when STAT5 was active [18], [24], [25]. Together these data support the idea that IL-21 (but not IL-2 in combination with IL-4) induces strong plasma cell differentiation, which can be prevented by activation of STAT5 [16], [18], [24], [25]. Interestingly, our B cell lines maintained the capacity to secrete immunoglobulin after prolonged culture (weeks to months) and even after freeze thawing (Figure S3).

### Establishment of cell lines from STAT5bER^pos^ memory B cells

Next we determined whether we could obtain stable cell lines from STAT5bER^pos^ memory B cells isolated from clonal density cultures. For this we isolated CD27^pos^ memory B cells from the peripheral blood of two different donors, cultured these cells with CD40L-L cells plus IL-21 for 36 hrs, transduced them with STAT5bER-IRES-GFP and subsequently cultured these cells with CD40L-L cells, IL-2, IL-4 and tamoxifen. After seven days GFP^pos^ B cells were plated out at a density of 1000, 100, 10 and 1 cell per well using high speed cell sorting and cultured with CD40L-L cells, IL-2, IL-4 and tamoxifen. Within two weeks multiple poly- and monoclonal STAT5bER memory B cell clones expanded. The frequency of outgrowth was 84%, 42%, 8% and 4% for the 1000, 100, 10 and 1 cell per well cultures respectively. These data show that stable STAT5bER^pos^ memory B cell clones can be obtained from clonal cultures.

### Isolation of Tetanus Toxin (TT)-specific STAT5bER^pos^ B cell lines

**Figure 3:**
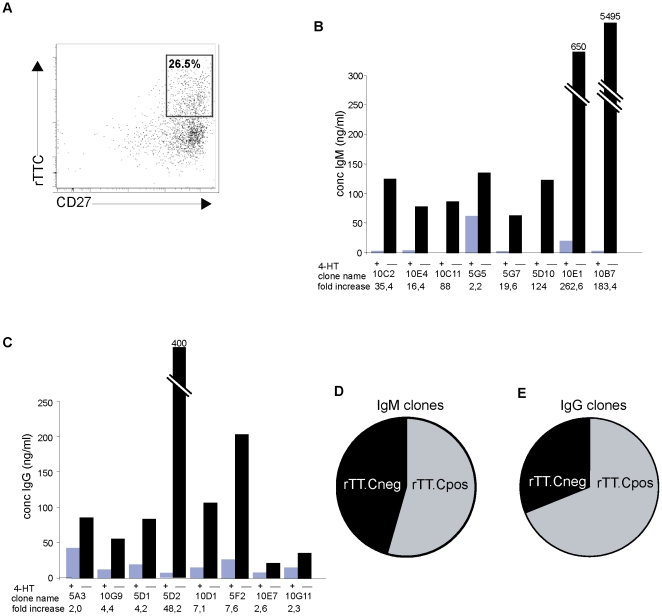
Generation of monoclonal rTT.C^pos^ STAT5bER^pos^ memory B cell lines. A) staining for rTT.C and CD27 on CD19 gated, CA-STAT5ER transduced peripheral blood B cells that were first sorted for rTT.C and CD27 before being transduced with STAT5bER. B) IgM and C) IgG ELISA performed on supernatants derived from the rTT.C^pos^ STAT5bER^pos^ memory B cell clones cultured with IL-2, IL-4 plus tamoxifen (4HT) or with IL-21 alone. Fold increase in secretion is shown. D) The frequency of rTT.C^pos^ clones within the IgM secreting clones (6 out of 11) and E) of IgG secreting clones (11 out of 16).

Since transduction of human B cells with STAT5bER can be utilized to develop immunoglobulin producing B cells lines cultured at clonal density, the next step was to determine whether we could make antigen specific immunoglobulin producing B cells lines. TT is produced by the gram-positive bacterium *Clostridium tetani*. Infants are routinely vaccinated with a combination vaccine against diphtheria, pertussis, tetanus, and poliomyelitis (DPTP) [26]. As a consequence, most adults have memory B cells specifically directed against TT in their blood that can be detected using PE-conjugated recombinant TT C-Fragment (rTT.C) (Figure S4) [15], [27]. Using flow cytometry rTT.C^pos^ CD27^pos^ memory B cells were isolated from the peripheral blood of healthy donors and cultured on CD40L-L cells in the presence of IL-21. After 36 hrs the cells were transduced with STAT5bER-IRES-GFP and cultured again on CD40L-L cells, now in the presence of IL-2, IL-4 and tamoxifen. Seven days after transduction the cells were checked for TT binding (Figure 3a), GFP sorted and maintained in microcultures of 1, 5 and 10 cells per well, again in the presence of CD40L-L cells, IL-2, IL-4 and tamoxifen. Within two weeks a number of rTT.C^pos^ STAT5bER^pos^ memory B cell clones could be selected (Table 1) and expanded further. Next we determined whether these rTT.C sorted cultures still had the capacity to produce immunoglobulins. After removal of tamoxifen from the cultures, rTT.C sorted STAT5bER^pos^ clones resumed production of either IgM or IgG (Figure 3b and c). Next we determined the antigen specificity of the rTT.C^pos^ STAT5bER^pos^ memory B cell lines. The majority of clones expressing either IgM (54%) or IgG (69%) were specific for TT as determined by IgM and IgG specific TT ELISA (Figure 3d and e). Together these data show that the immortalizing capacity of STAT5bER can be used to obtain B cell lines with the capacity to secrete antigen specific immunoglobulins.

**Table 1:** Limiting dilution culture of 100% GFP^pos^ TT-specific B cells.

donor	Number of positive clones from 96 wells			
	Total #	from 1 c/w	from 5 c/w	from 10 c/w
A	12/96	2	10	N.D.
B	14/96	0	7	7
C	10/96	10	0	0
D	11/96	1	3	7

Indicated is the total number of clones isolated and at which cell density they were seeded. One 96 well plate was used for each condition (1, 5 or 10 cell per well). Wells contained 2500 CD40L-expressing L cells, IL-2 and IL-4. ¼ to ½ of the medium was replaced twice a week with fresh cytokines and 5000 L cells. N.D. is not determined.

It should be mentioned that we also found high numbers of IgA expressing cells in our STAT5bER transduced memory B cell populations, which indicates that these IgA expressing cells can be transduced and maintained in culture like the IgG and IgM expressing cells (Figure S5).

### rTT.C sorted STAT5bER^pos^ clones are monoclonal and have undergone somatic hypermutation

**Figure 4:**
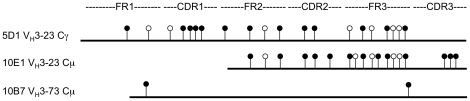
Schematic representation of IgV_H_ consensus sequence of TT^pos^ STATbER^pos^ memory B cell clones; 5D1, 10E1 and 10B7. The horizontal bar of each clone represents the sequenced region. The FR1 to CDR3 region was sequenced for clone 5D1 and for clones 10E1 and 10B7 region FR2 to CDR3 were sequenced. We were unable to obtain reliable sequence data of the FR1- CDR1 region of clone 1E10. The lollipop-shaped symbols indicate nucleotide difference with germline configuration, except for the CDR3 region. The CDR3 region of 10E1 is compared to 5D1. Replacement and silent mutations are indicated by closed and open circles, respectively.

A crucial hallmark of memory B cells is that the cells have undergone somatic hypermutation [28] and affinity maturation, the mechanisms via which they become able to develop high affinity antibodies. For this reason we determined the mutation status of TT specific STAT5bER^pos^ memory B cell lines from one donor (Figure 3d and e). Monoclonality of memory B cell lines that were cultured at clonal density (Table 1) could be confirmed by VDJ sequencing per cell line, as only one VDJ sequence per cell line was detected (Figure 4). From this one donor, we obtained three independent immunoglobulin sequences – one IgG and two IgM sequences (Figure 4). Interestingly, one IgG and one IgM had a similar basic pattern of somatic hypermutation, with the IgM sequence containing 6 additional mutations, one of which led to an amino acid change. This suggests that these memory B cells arose from the same clone, and that the IgM clones had undergone additional somatic hypermutation and affinity maturation.

## Discussion

The results presented in this study demonstrate that the immortalizing capacity of an inducible active STAT5 mutant can be used to obtain human, antigen-specific, monoclonal B cell lines, which have the capacity to undergo plasma cell differentiation. After turning off STAT5 these cells can then be induced to secrete immunoglobulins. By making use of memory B cells isolated from healthy donors we could screen for B cells that have been positively selected for high affinity against a specific antigen.

Recently we published evidence that continued activation of STAT5 plays a role in the lymphomagenesis of classical Hodgkin Lymphoma [17]. We showed that long-term culture of primary B cells expressing constitutive active STAT5 mutants eventually led to a phenotype closely resembling classical Hodgkin Lymphoma cells, including the loss of immunoglobulin expression and other B cell specific markers as CD20 [29], [30], [31]. The absence of immunoglobulin expression is an important hallmark of classical Hodgkin lymphoma [32]. In approximately 25% of the cases mutations leading to a crippled immunoglobulin expression can be found [32], [33]. However, in the majority of the cases no crippled mutations can be found and epigenetic events on the promoter region of the immunoglobulin heavy chain have been proposed as an alternative explanation for the absence of immunoglobulin expression by Hodgkin Lymphoma cells [28].

In this paper we show that inactivation of STAT5 (by removing tamoxifen from the cultures) in the presence of IL-21 results in re-appearance of immunoglobulin expression, although levels are still relatively low compared to levels normally described for *ex vivo* derived plasma cells. This suggests that the loss of immunoglobulin expression by primary B cells transduced with STAT5bER is dependent on activation of STAT5. We have found previously that activation of STAT5 in primary B cells inhibits the expression of Blimp-1. Blimp-1 is a transcription factor crucial for plasma cell differentiation [16], [34], [35], and we here postulate that inactivation of STAT5 in our cell cultures leads to re-activation of Blimp-1 and subsequent differentiation of these cells into antibody secreting plasma cells.

The STAT5b-ER transduced B cells only secrete antibody when STAT5b-ER is inactivated and in the presence of IL-21. IL-21 induces proliferation and plasma cell differentiation in B cells, both in mice as well as in humans [23], [24], [25], [36], [37]. This occurs at least partly through upregulation of Blimp-1 by STAT3 activation [18], [38], [39]. IL-21 can also activate STAT5 and thus counteract STAT3 induced differentiation but the activation of STAT5 by IL-21 is only transient (30 hrs) compared to STAT3 (days). Stimulation with IL-2 and IL-4 did not lead to antibody secretion, which is expected since both cytokines induce proliferation but do not plasma cell differentiation of B cells [16], [40], [41], [42].

The extended lifespan induced by forced expression of inducible STAT5 mutants described in this paper provides a tool to expand *in vitro* cultured single human memory B cells to obtain monoclonal cell lines. However these cell lines do not express any immunoglobulins when the inducible active STAT5 mutant is “on”. By subsequently inactivating STAT5 the cells regained the capacity to produce immunoglobulin offering an opportunity to screen the clones for secretion of specific antibodies. It should be noted that antigen specificity was not selected for by a functional assay. Instead, we used a binding assay (ELISA) to screen for anti-tetanus toxoid specific antibodies. It has been described previously however that TT specific antibodies selected through binding assays (ELISA) are functional as shown in a Toxin Binding Inhibition test (ToBI) and in *in vivo* TT challenge models in mice [43] [44]. Following selection of the antigen-specific clones the immunoglobulin genes can be retrieved from the clones. An obvious next step would be to scale up the production of antibodies through recombinant expression of the identified immunoglobulin genes in suitable cell lines. These recombinant antibody proteins can then be tested for other effector functions like antigen dependent cellular cytotoxicity (ADCC), complement activation or *in vivo* in mice for toxin inactivation properties.

The relevance of the technology we present here is that it can be used to obtain monoclonal antibodies that are antigen specific, even when present only in low frequencies in humans. Indeed, direct isolation from the blood of influenza virus-specific B cells with a highly restricted B cell receptor repertoire has been described recently. However, healthy volunteers in this study had received booster vaccination against Influenza, inducing high percentages of antibody secreting plasma cells in the peripheral blood of these individuals [12]. Indeed, such abundance of antigen-specific plasma cells is unique. Much more often the frequency of antigen specific memory B cells is low, for example in cases where appropriate vaccines to induce such overt antibody response are lacking. In those instances the method described here may be applied to isolate human antigen specific monoclonal antibodies.

## Materials and Methods

### Ethics Statement

The use of human materials was approved by the Medical Ethical Committee of the Academic Medical Center of the University of Amsterdam (project MEC 07/248) and was contingent on written informed consent.

### B cell isolation

B cells were obtained from buffy coats prepared from the peripheral blood of adults (Sanquin, Amsterdam, Netherlands) by Ficoll separation and CD19 MACS microbeads as described by the manufacturer (Miltenyi Biotech). CD19 MACS-selected B cells were subsequent sorted for CD3^neg^CD19^pos^CD27^pos^, CD3^neg^CD19^pos^CD27^pos^sIgG^pos^ or CD3^neg^CD19^pos^CD27^pos^ TT^pos^.

### Flow cytometry

The following mAbs against the human molecules CD3 (SK7), CD19 (SJ25C1), IgG (G18-145) (BD-Pharmingen, San Diego, CA), CD27 (O323; eBioscience) and IgA (F(ab)_2_, DAKO), were directly labeled with either fluoroscein isothiocyanate (FITC), phycoerythrin (PE), phycoerythrin cyanine 5, (PE-Cy5), allophycocyanin (APC), phycoerythrin-indotricarbocyanine (PE-Cy7), phycoerythrin-cyanine 7 (PC7) or allophycocyanin-indotricarbocyanine (APC-Cy7). Recombinant TT C-fragment was purchased from Boehringer Mannheim (Mannhein, Germany) and conjugated to PE (Cyanotech Corporation, Kaila-kona, HI) as previously described [27]. Stained cells were sorted using a FACSAria (BD) and analyzed using a LSR-II (BD) and flow cytometry data were processed with FlowJo computer software (Treestar, Ashland, OR).

### Retroviral transduction

The STAT5bER retroviral construct has been described previously [16]. In brief, cDNA encoding STAT5bER was expressed in the Lazarus (LZRS) vector upstream to a internal ribosomal entry site (IRES) and a marker gene (a signaling-incompetent mutant of NGFR; provided by C. Bonini, St. Raphael Hospital, Milan, Italy or GFP) that allow independent translation of the products of both genes in the transduced target cells. Retroviral production by Phoenix packaging cells were performed as described earlier [16]. B cells were transduced with retroviruses after activation on CD40L-L cells in the presence of recombinant mouse IL-21 (25 ng/ml; R&D systems) for 36 hrs. After the transductions, cells were cultured in the presence of IL-2 and IL-4 (R&D systems).

### Cell culture

B cells (2×10^5^) were co-cultured on γ-irradiated (100 Gy) mouse L cell fibroblasts stably expressing CD40L (CD40L-L cells, 10^5^ well) and recombinant human IL-2, IL-4 or IL-21. Cells were routinely tested by PCR for the presence of mycoplasma and EBV (data not shown).

### ELISA

Plates were coated with anti-human IgG (Dako) at 5 µg/ml in PBS or Tetanus vaccine (Dutch vaccine institute, The Netherlands) diluted 1∶10 for 1 hr at 37°C or o/n at 4°C and washed in ELISA wash buffer (PBS, 0.5% Tween-20). 4% milk in PBS was used as blocking agent, before serial dilution of cell culture supernatants and enzyme-conjugated detection Abs were added (dilutions 1∶2500 for HRP-conjugated anti-IgG (Jackson ImmunoResearch Laboratories) and 1∶250 for AP conjugated anti-IgG (DAKO)). TMB substrate/stop solution (Biosource) or Alkaline Phosphatase Substrate (Sigma-Aldrich) was used for development of the ELISAs. To detect Tetanus Toxin we used the ELISA Ridascreen Tetanus (r-biopharm AG, Darmstadt, Germany).

### Somatic hyper mutation analysis

Total RNA was isolated from approximately 5×10^5^ B cells with Trizol (Invitrogen). cDNA was generated and subjected to PCR to produce heavy chain fragments using 1 U AmpliTaq Gold DNA polymerase (Applied Biosystems Inc.). PCR products were run on agarose gels, purified and cloned into the pCR2.1 TA cloning vector according to manufacturers' recommendations (Invitrogen). Sequence analysis was performed using BigDye Terminator chemistry (Applied Biosystems Inc.) and Vector-NTI software (Invitrogen). Consensus sequences were determined with Codon code (CodonCode Corporation).

## Supporting Information

Figure S1 **IL-21 induced Ig secretion in STAT5bER immortalized memory B cell cultures.** A) STAT5bER transduced polyclonal memory B cells were cultured in the absence of tamoxifen (4HT) and with IL-2 and IL-4 or IL-21. IgG secretion was determined at day 7. Data from two representative donors out of 5 donors is shown. B) STAT5bER transduced monoclonal memory B cell lines were cultured in the absence of tamoxifen (4HT) and with IL-2 and IL-4 or IL-21. IgG secretion was determined on day 7. Data from two representative cell cultures is shown. All B cells in these cultures had lost surface immunoglobulin expression and were in culture for at least 2 months.
(TIF)

Figure S2 **IgG and IgM secretion in time after removal of tamoxifen (4HT).** IgG and IgM secretion was determined in cells cultured with CD40L and IL-21 for 7 days and for 14 days. Days 0 indicates the day when the culture conditions were changed from CD40L, IL-2, IL-4 plus tamoxifen (4HT) to CD40L and IL-21. The black and grey bars represents two different donors.
(TIF)

Figure S3 **IgG and IgM expression in long term cultures.** Multiple TT specific clones which were in culture for more then 5 months and subsequent frozen. They were then thawed and taken in culture again with CD40L, IL-2, IL-4 and tamoxifen (4HT). When a stable culture was obtained, the cells were cultured with CD40L and IL-21. An ELISA was performed to determine IgG and IgM concentrations.
(TIF)

Figure S4 **rTT.C staining on freshly isolated memory B cells.** Phycoerythrin (PE) labeled rTT.C was added to freshly isolated B cells. Cells were subsequently sorted using flow cytometry (FACSAria). Shown are the results of 5 different donors.
(TIF)

Figure S5 **IgA and IgG expression on polyclonal CA-STAT5b transduced and non-transduced B cells.** A polyclonal mixture of total CD27+ selected human memory B cells from two donors were transduced with caSTAT5b-IRES-NGFR and cultured for a maximum of two weeks before they were frozen. After thawing cells were stained immediately for the IgA and IgG isotype. Cells expressing NGFR indicates they were transduced with CA-STAT5b.
(TIF)
