# Supplementary figures and images for: Antigen-Specific Monoclonal Antibodies Isolated from B Cells Expressing Constitutively Active STAT5
Source: PLoS One. 2011 Apr 15;6(4):e17189. doi: 10.1371/journal.pone.0017189 (PMC3078118; doi:10.1371/journal.pone.0017189)

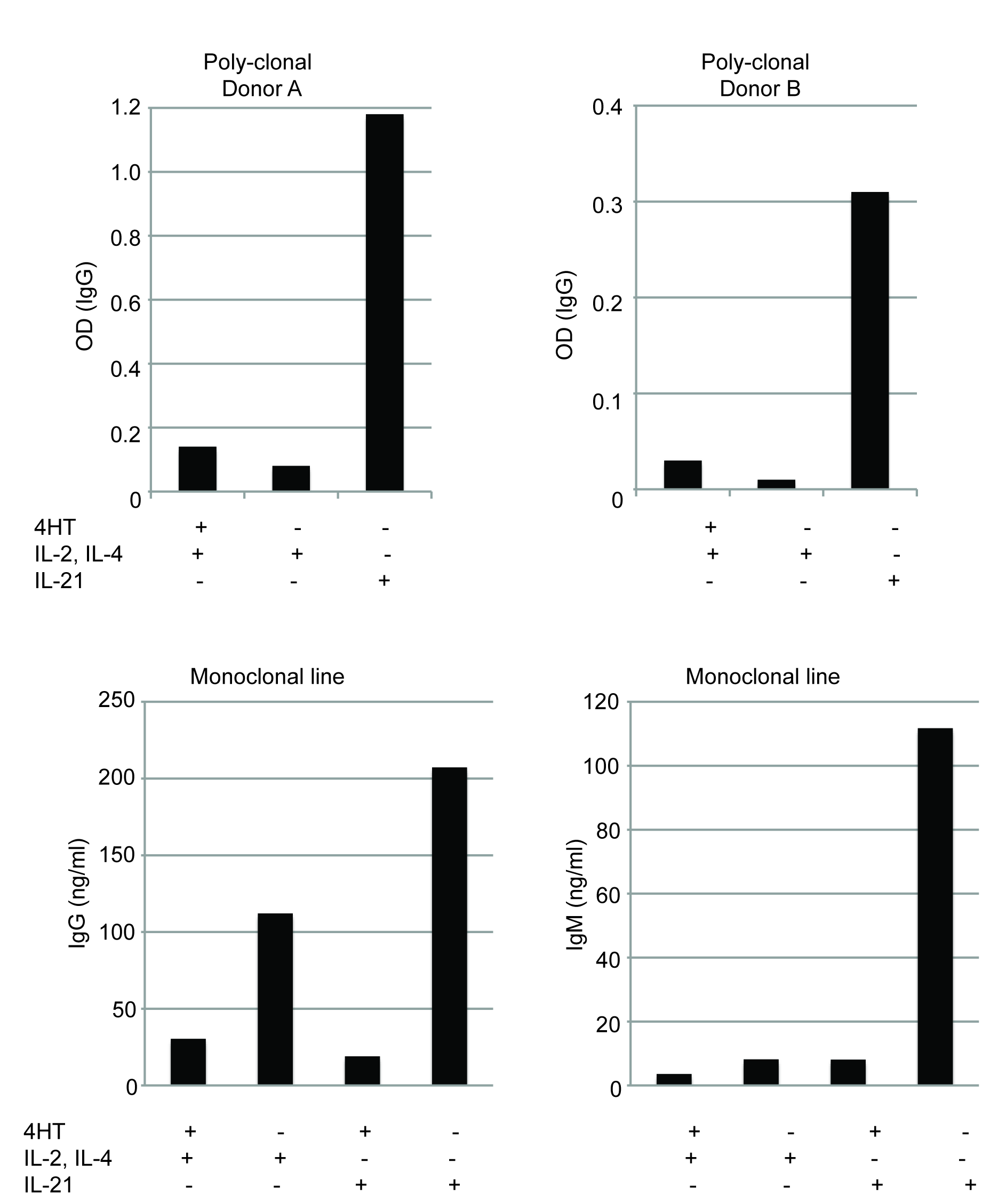

Supplement: Figure S1 — IL-21 induced Ig secretion in STAT5bER immortalized memory B cell cultures. A) STAT5bER transduced polyclonal memory B cells were cultured in the absence of tamoxifen (4HT) and with IL-2 and IL-4 or IL-21. IgG secretion was determined at day 7. Data from two representative donors out of 5 donors is shown. B) STAT5bER transduced monoclonal memory B cell lines were cultured in the absence of tamoxifen (4HT) and with IL-2 and IL-4 or IL-21. IgG secretion was determined on day 7. Data from two representative cell cultures is shown. All B cells in these cultures had lost surface immunoglobulin expression and were in culture for at least 2 months. (TIF) [file pone.0017189.s001.tif]

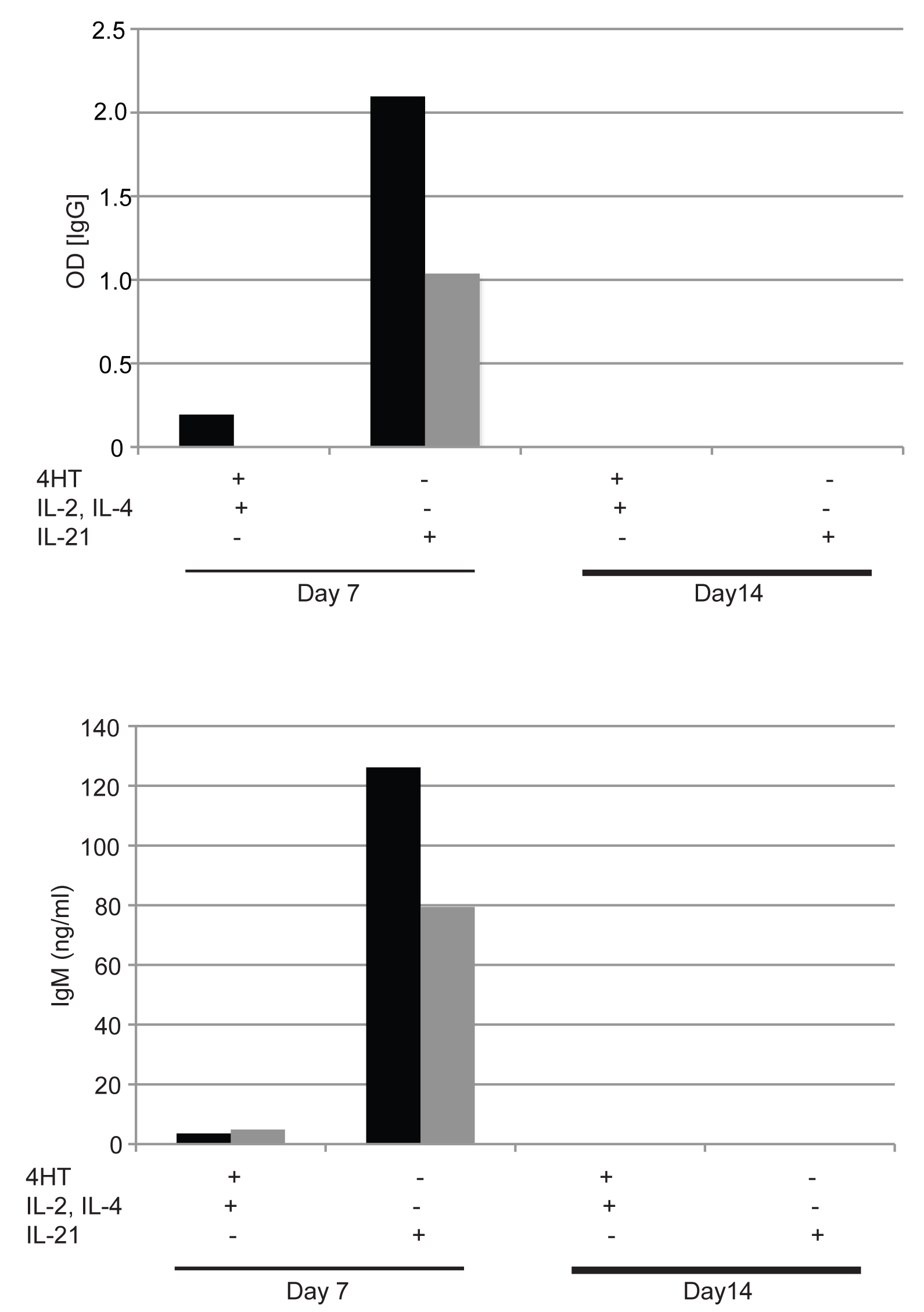

Supplement: Figure S2 — IgG and IgM secretion in time after removal of tamoxifen (4HT). IgG and IgM secretion was determined in cells cultured with CD40L and IL-21 for 7 days and for 14 days. Days 0 indicates the day when the culture conditions were changed from CD40L, IL-2, IL-4 plus tamoxifen (4HT) to CD40L and IL-21. The black and grey bars represents two different donors. (TIF) [file pone.0017189.s002.tif]

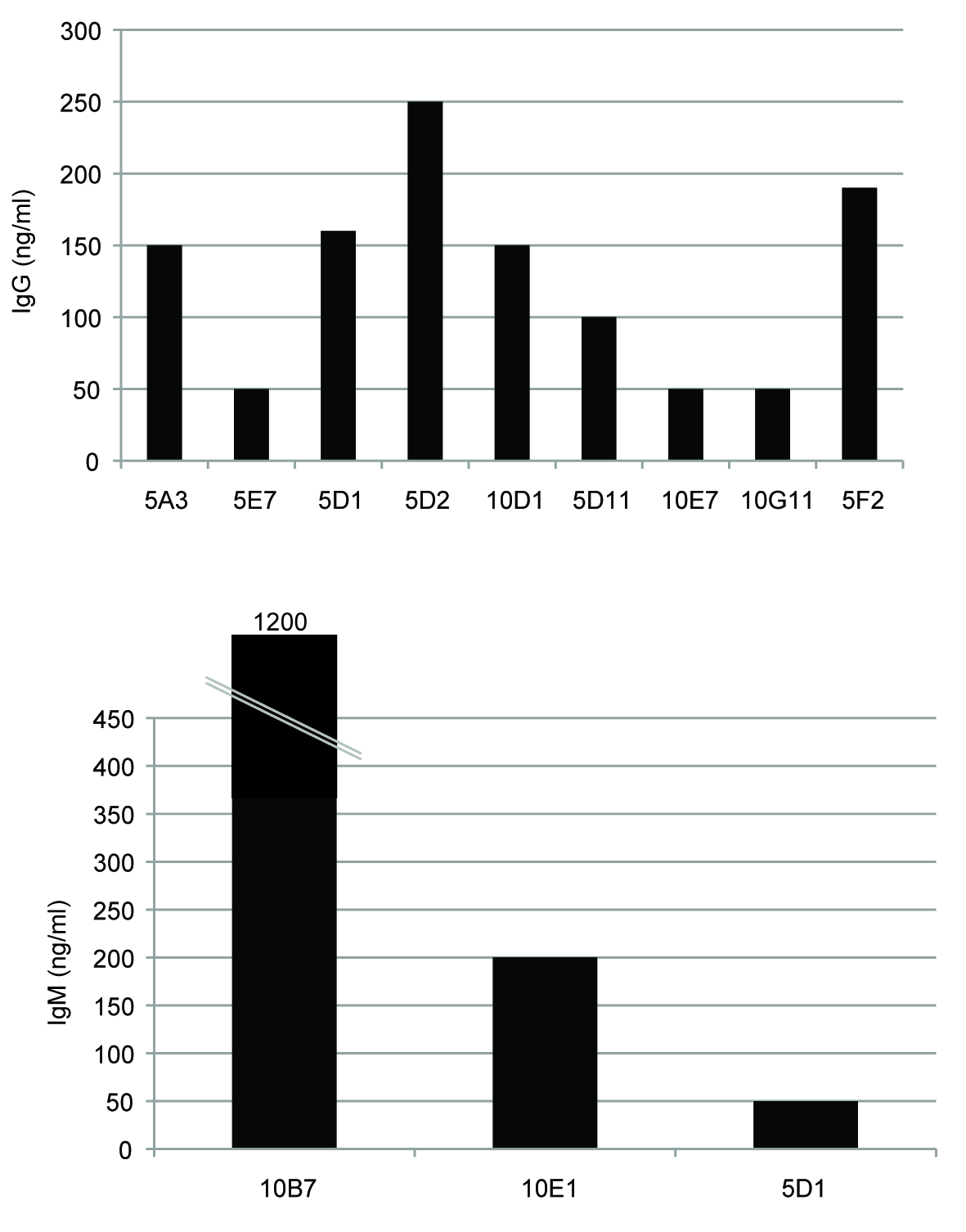

Supplement: Figure S3 — IgG and IgM expression in long term cultures. Multiple TT specific clones which were in culture for more then 5 months and subsequent frozen. They were then thawed and taken in culture again with CD40L, IL-2, IL-4 and tamoxifen (4HT). When a stable culture was obtained, the cells were cultured with CD40L and IL-21. An ELISA was performed to determine IgG and IgM concentrations. (TIF) [file pone.0017189.s003.tif]

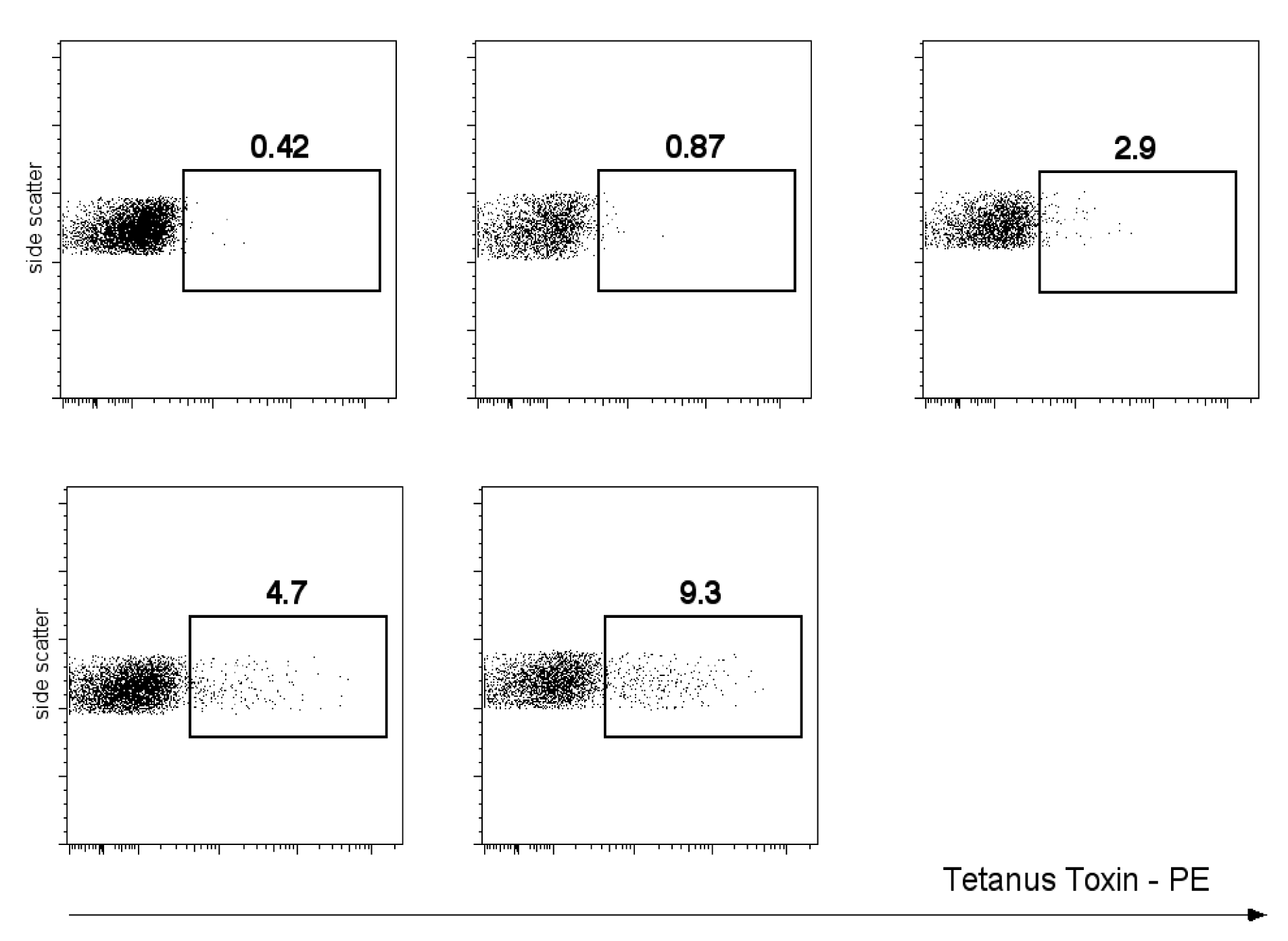

Supplement: Figure S4 — rTT.C staining on freshly isolated memory B cells. Phycoerythrin (PE) labeled rTT.C was added to freshly isolated B cells. Cells were subsequently sorted using flow cytometry (FACSAria). Shown are the results of 5 different donors. (TIF) [file pone.0017189.s004.tif]

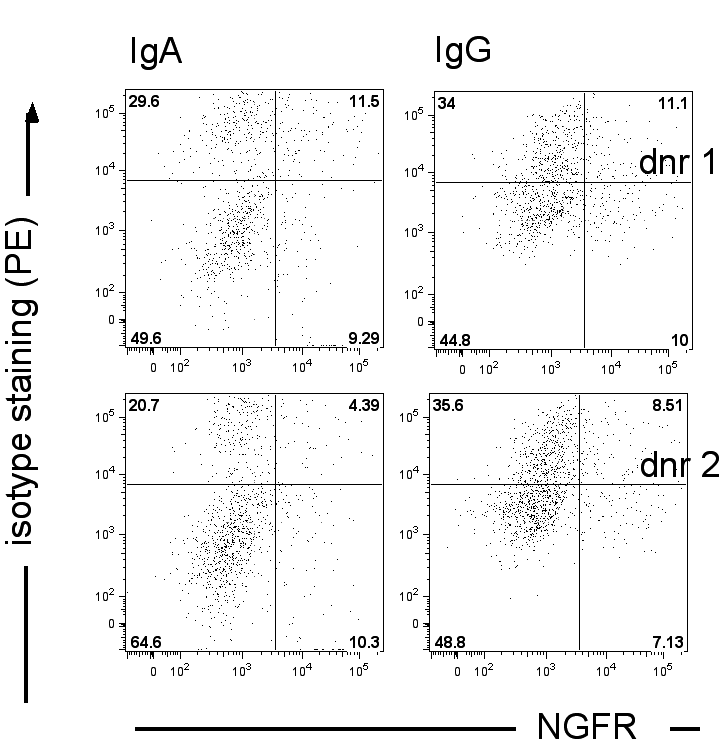

Supplement: Figure S5 — IgA and IgG expression on polyclonal CA-STAT5b transduced and non-transduced B cells. A polyclonal mixture of total CD27+ selected human memory B cells from two donors were transduced with caSTAT5b-IRES-NGFR and cultured for a maximum of two weeks before they were frozen. After thawing cells were stained immediately for the IgA and IgG isotype. Cells expressing NGFR indicates they were transduced with CA-STAT5b. (TIF) [file pone.0017189.s005.tif]
